# Denys‐Drash Syndrome by *WT1* Gene: Clinical Variability and Management Challenges in Two Saudi Infants

**DOI:** 10.1155/crie/8875066

**Published:** 2026-04-24

**Authors:** Waleed AL-Amoudi, Raghad Alhuthil, Abdulrahman Alnwiji, Megren S. Alqarni, Sarah Murad, Afaf Alsagheir

**Affiliations:** ^1^ College of Medicine, Alfaisal University, Riyadh, Saudi Arabia, alfaisal.edu; ^2^ Department of Pediatrics, King Faisal Specialist Hospital and Research Centre, Riyadh, 11211, Saudi Arabia, kfshrc.edu.sa; ^3^ Department of Radiology, King Faisal Specialist Hospital and Research Centre, Jeddah, Saudi Arabia, kfshrc.edu.sa

**Keywords:** ambiguous genitalia, Denys-Drash syndrome, Wilms tumor, *WT1* gene

## Abstract

**Background:**

Denys‐Drash syndrome (DDS) is a rare genetic disorder characterized by mutations in the Wilms tumor suppressor gene (*WT1*), leading to a triad of conditions including nephrotic syndrome progressing to end‐stage renal disease (ESRD), Wilms tumor, and ambiguous genitalia. We present two pediatric cases illustrating the complexity and variability of DDS.

**Case presentation:**

The first case is a 7‐month‐old male presenting with ambiguous genitalia, nephropathy, and intussusception. Genetic analysis identified a likely pathogenic heterozygous *WT1* variant: c.1384C >T (p.Gln462Ter). Despite surgical interventions, treatment was delayed due to COVID‐19 restrictions, and the patient unfortunately passed away at 16 months during the lockdown period. The second case involves an 8‐month‐old female with normal external genitalia, a horseshoe kidney, bilateral renal masses, and recurrent hypotensive episodes. Genetic testing revealed a pathogenic heterozygous *WT1* variant: c.453G >A (p.Trp151Ter). She was diagnosed with DDS–associated Wilms tumor and, despite aggressive management, passed away at 21 months.

**Conclusion:**

These cases underscore the importance of early diagnosis, multidisciplinary management, and personalized therapeutic approaches in DDS patients. Bilateral nephrectomy, renal transplantation, and monitoring for Wilms tumor are pivotal in improving prognosis, though variability in clinical presentations often complicates decision‐making.

## 1. Introduction

Denys‐Drash syndrome (DDS) is part of the broader spectrum of WT1–related disorders (WT disorders), a group of rare genetic conditions caused by pathogenic variants in the Wilms tumor suppressor gene (WT1), which plays an essential role in the development of the renal and genital systems [1]. The gene is located on chromosome 11, with pathogenic variants frequently occurring in exons 8 and 9 [1, 2]. DDS is classically defined by a triad of early‐onset nephrotic syndrome progressing to end‐stage renal disease (ESRD), Wilms tumor (nephroblastoma), and ambiguous genitalia [1]. However, the clinical spectrum of WT1–related disorders extends beyond classic DDS and includes overlapping phenotypes such as DDS, Frasier syndrome, and steroid‐resistant nephrotic syndrome type 4 (NPHS4). These conditions are characterized by varying combinations of nephropathy, gonadal dysgenesis, and predisposition to Wilms tumor. Significant phenotypic overlap exists among these entities; in some patients renal failure may develop before tumor formation, whereas in others Wilms tumor may occur prior to the onset of nephropathy [2].

Furthermore, sexual development in DDS varies significantly; males may present with ambiguous genitalia or normal male phenotype, whereas females typically have normal external genitalia [3]. Importantly, management is quite challenging due to the variability in both the clinical course and evolution of the disease; however, management generally follows two main strategies: prophylactic bilateral nephrectomy prior to progression to irreversible renal failure, Wilms’ tumor, and decreasing the total duration of dialysis or performing bilateral nephrectomy after already progression of nephropathy to ESRD [1, 4, 5]. On the other hand, there are sets of patients managed conservatively; those patients will be followed by surveillance in which serial imaging studies can be used and hence detect the emergence of Wilms tumor [1, 6]. As Wilms’ tumor arose, some cases underwent bilateral nephrectomy while others underwent nephron‐sparing surgery to conserve renal function as much as possible [1, 7].

Ambiguous genitalia refers to a condition categorized under differences of sex developmentin which the appearance of the external genitalia is atypical [8]. The prevalence of ambiguous genitalia is estimated to be around one in 5000 live births [9]. Sexual differentiation of the external genitalia depends on various factors, including genetic sex, gonadal sex, or androgen action [10]. Disruption of these factors can lead to abnormal external genitalia development [11]. This is observed in conditions such as DDS, congenital adrenal hyperplasia, and androgen insensitivity syndrome [12].

Given the psychological and clinical complexity, the management of infants with ambiguous genitalia requires prompt and sensitive evaluation. A multidisciplinary approach involving pediatric nephrology, oncology, endocrinology, urology, genetics, and specialists with expertise in disorders of sex development (DSD) is essential. The presence of DSD expertise is particularly important when counseling families and making decisions regarding gonadal preservation or prophylactic gonadectomy. Collaborative efforts with the family aim to establish an accurate diagnosis, guide gender assignment, and optimize both short‐ and long‐term outcomes [13].

## 2. Case Presentation

### 2.1. Case 1

A 7‐month‐old male infant known case of congenital hypothyroidism on levothyroxine was referred as a case of ambiguous genitalia for further investigations. The pregnancy was uneventful, and the parents were healthy, unrelated, but from the same tribe. There was no family history of similar conditions, and the infant had three healthy siblings.

Prenatal ultrasound detected a cyst behind the bladder, with identified gonads. After birth, it was noted that the baby had a small phallus, hypospadias, fused labioscrotal fold, and no palpable gonads. Karyotyping was done, and it was found that the baby was a male (46XY; Table 1).

**Table 1:** Summary of demographics, genetics, and clinical findings of the two cases.

Parameter	Case 1 (male, 46XY, 7 months)	Case 2 (female, 46XX, 8 months)
Clinical presentation	Mixed gonadal dysgenesis, hypothyroidism, recurrent infections, heat intolerance, hypertension, acute kidney injury, ileocolic intussusception. No dysmorphic features	Recurrent hypotensive attacks, bilateral palpable flank masses, abdominal distension, normal genitalia. No dysmorphic features
Family history	Negative for DDS and DSD disorders	Negative for DDS and DSD disorders
Growth data	Height: 60 cm (−2.8 SDS), weight: 6 kg (−2.4 SDS)	Height: 65 cm (−1.52 SDS), weight: 6.4 kg (−2.30 SDS)
Laboratory findings	‐High urea: 12.9 mmol/L (ref. 2.5–6.5) ‐High creatinine: 77 µmol/L (ref. 18–48) ‐Cotisol: 86.9 nmol/L (ref. 83–414) ‐High ACTH: 12.3 ng/L (ref. 5–60) ‐17‐Hydropregestrone <0.4 nmol/L (ref. <3.3) ‐Testosterone <0.100 nmol/L (ref. 0.42–0.72) ‐17‐Hydroxypregnelonone: <16 ng/dL (ref. 221–1981) ‐Low FT4: 9.7 pmol/L (ref. 12–22), ‐High TSH: 17.100 mU/L (ref. 0.270–4.200) ‐High PTH: 331.0 ng/L (ref. 15–65) ‐High ALP: 172 U/L (ref. 122–469)	‐High urea: 15.8 mmol/L (ref. 2.5–6.5) ‐High creatinine: 191 µmol/L (ref. 18–48) ‐Cortisol: 399.0 nmol/L (ref. 83–414) ‐High ACTH: 123 ng/L (ref. 5–60) ‐High TSH: 15.100 mU/L (ref. 0.270–4.200) ‐FT4: 15.1 pmol/L (ref. 12–22) ‐High PTH: 390.0 ng/L (ref. 15–65) ‐High ALP: 823 U/L (ref. 122–469)
Imaging findings	Renal US: enlarged kidneys, increased echogenicity, poor corticomedullary differentiation, moderate left hydronephrosis, bilateral interpolar cystic lesions with minimal vascularity CT/MRI: confirmed renal findings	Renal US: large bilateral lower pole masses with central hypoechoic/necrotic center and minimal vascularity CT: horseshoe kidney with bilateral heterogeneous enhancing soft tissue masses
Diagnosis	Mixed gonadal dysgenesis, ambiguous genitalia, hypothyroidism, acute kidney injury, ileocolic intussusception	Stage 4 Wilms tumor (*WT1* mutation), chemotherapy‐induced cardiomyopathy
Treatment	Levothyroxine, steroids, antihypertensives, calcium carbonate, epoetin alfa, ferrous sulfate, antibiotics, lactulose, alfacalcidol, sodium chloride. Planned bilateral nephrectomy, but patient passed away before surgery	Aggressive chemotherapy (doxorubicin), bilateral radical nephrectomy, peritoneal dialysis. Multiple PICU admissions for sepsis, respiratory failure, and cardiomyopathy
Outcome	Developed end‐stage renal disease, nephrotic syndrome. Passed away at 16 months during COVID‐19 lockdown	Passed away at 21 months in PICU due to septic shock, respiratory failure, and multiorgan dysfunction. Transitioned to comfort care (DNAR signed) before cardiopulmonary arrest

Abbreviations: ACTH, adrenocorticotropic hormone; ALP, alkaline phosphatase; DDS, Denys‐Drash syndrome; DNAR:, do not attempt resuscitation; DSD, disorders of sex development; FT4, free thyroxine; HLH, hemophagocytic lymphohistiocytosis; PTH, parathyroid hormone; SDS, standard deviation score; TSH, thyroid‐stimulating hormone; US, ultrasound; VUS, variant of uncertain significance.

**Figure 1:**
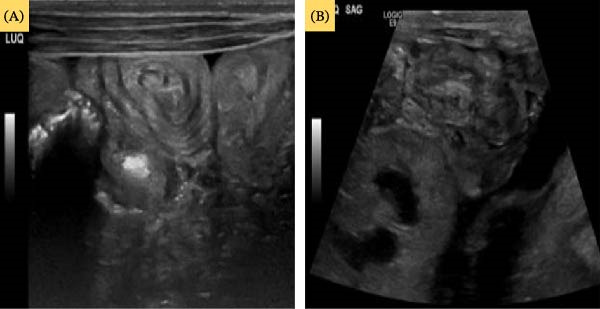
Targeted ultrasound to the right lower abdomen showing (A) target sign and (B) pseudo‐kidney sign, suggestive of intussusception in the terminal ileum (Case 1).

At 1 month of age, the patient underwent surgery in which the bladder was connected to the abdominal wall. For the next 2 months, the patient was stable until he developed hypertension and was diagnosed to have acute kidney injury due to elevated creatinine and urea levels. He also presented with bloody stool, which was later found to be due to ileocolic intussusception (Figure 1). Partial reduction using contrast enema was performed, followed by manual reduction by the pediatric surgeon. At the time of referral to our hospital, his examination revealed no dysmorphic features; however, there was hyperpigmentation in the lower limbs with no edema. Central nervous system (CNS) examination was unremarkable. On auscultation, normal S1 and S2 were heard. Lungs showed clear air entry bilaterally. The abdomen was mildly distended, no organomegaly was noted; however, the right kidney was palpable. Genital exam showed ambiguous hypospadias, a small phallus, perineal hypospidus, scrotum not fully developed, and a normal patent anus. The patient was on the following medications: amlodipine 2.5 mg orally once daily, labetalol 10 mg orally twice daily, and levothyroxine 50 mcg orally once daily.

**Figure 2:**
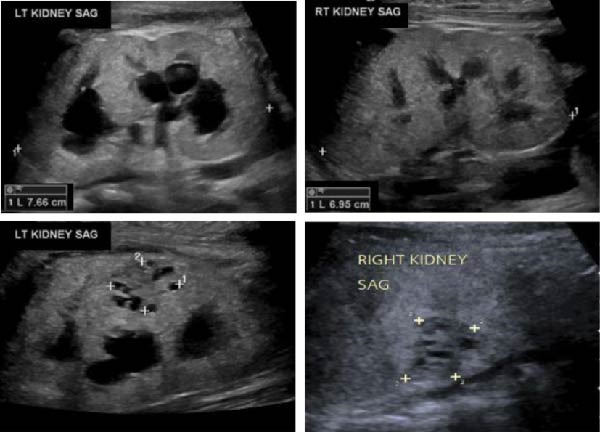
Renal ultrasound showing bilateral enlarged echogenic kidneys with loss of corticomedullary differentiation. Left moderate hydronephrosis. Bilateral cystic, solid lesions in the interpolar region with minimal internal vascularity in Color Doppler Images (not shown; Case 1).

**Figure 3:**
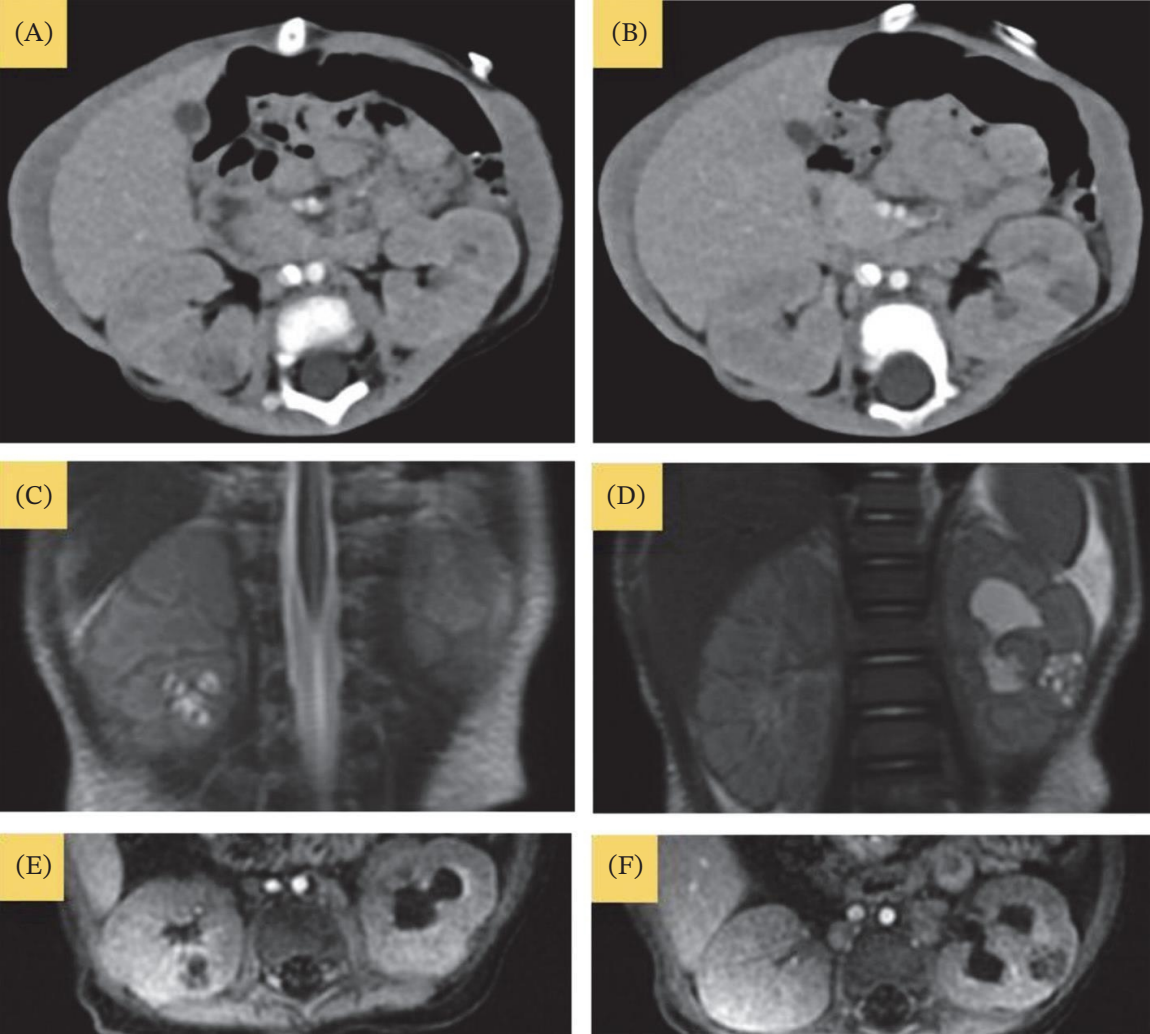
(A, B) Enhanced axial CT scan of the abdomen at the level of kidneys showing bilateral interpolar complex cystic solid lesions. (C, D) T2WI showing bilateral multiseptated cystic lesions. (E, F) T1 postcontrast and coned‐down view of the kidneys shows early homogeneous septa and periphery enhancement. Left moderate hydronephrosis is also appreciated (Case 1).

Renal ultrasound (Figure 2) showed enlarged kidneys with increased echogenicity and poor corticomedullary differentiation. Left‐moderated hydronephrosis was noted. A complex solid cystic lesion was seen in the bilateral interpolar region with minimal internal vascularity. Similar findings were appreciated on abdominal computed tomography (CT) and renal magnetic resonance imaging (MRI; Figure 3).

Genetic testing was performed using single‐gene next‐generation sequencing (NGS) of the WT1 gene, which identified a heterozygous nonsense variant c.1384C >T (p.Gln462Ter; Table 2). According to the American College of Medical Genetics and Genomics (ACMG) criteria, this variant fulfills PVS1 (null variant in a gene where loss of function is a known disease mechanism), PM2 (absence or extremely low frequency in population databases), and PP3 (supporting computational evidence). The variant was confirmed by Sanger sequencing in the index patient, and parental testing was negative, supporting a de novo occurrence.

**Table 2:** Details of the genetic variants identified.

Case	Zygosity	Gene	Exon	Transcript ID	Genomic location (GRCh38)	Variant (HGVS, cDNA)	Protein change	gnomAD population frequency	ACMG criteria	ACMG class	dbSNP
Case1	He	*WT1*	9	NM_024426.6	chr11:32392035	SNV (nonsense), c.1384C >T	p.(Gln462Ter)	0% absent in controls (PM2): strong evidence supporting pathogenicity if all other criteria align	‐PVS1 ^∗^ ‐PM2 ^∗∗^ ‐PP3 ^∗∗∗^	LP	rs2132914929
Case 2	He	*WT1*	1	NM_024426.6	chr11:32434908	SNV (nonsense), c.453G >A	p.(Trp151Ter)	Not reported	‐PVS1 ^∗^ ‐PM2 ^∗∗^ ‐PP3 ^∗∗∗^	P	rs1267712523

*Note:* Variants were annotated using transcript NM_024426.6 based on the GRCh38 (hg38) genome build. gnomAD, Genome Aggregation Database; dbSNP: Single Nucleotide Polymorphism Database; GRCh38 (hg38), Genome Reference Consortium Human Genome build 38.

Abbreviations: ACMG, American College of Medical Genetics and Genomics; AF, allele frequency; Het, heterozygous; HGVS, Human Genome Variation Society; LP, likely pathogenic; P, pathogenic; SNV, single nucleotide variant.

^∗^PVS1 = predicted null variant in a gene where loss of function is a known disease mechanism.

^∗∗^PM2 = absent or extremely rare in population databases.

^∗∗∗^PP3 = multiple computational tools support a deleterious effect.

Consequently, the patient developed ESRD due to congenital nephrotic syndrome and was started on peritoneal dialysis. A bilateral nephrectomy was planned, but he passed away at 16 months old during the COVID‐19 lockdown (March 2020).

### 2.2. Case 2

An 8‐month‐old female, born full‐term via spontaneous vaginal delivery and discharged in good health, presented with abdominal distension following a recent episode of diarrhea and fever (38.8°C), along with recurrent hypotensive episodes. On examination, she was alert, oriented, afebrile, and well‐hydrated. However, bilateral palpable abdominal masses were noted over the flank regions. The remainder of the systemic examination was unremarkable.

Karyotyping confirmed a normal female genotype (46, XX; Table 1). Whole‐exome sequencing (WES) identified a heterozygous nonsense WT1 variant c.453G >A (p.Trp151Ter; Table 2). According to ACMG guidelines, this variant fulfills PVS1, PM2, and PP3 criteria, consistent with pathogenic classification. The variant is absent or extremely rare in population databases including gnomAD, further supporting pathogenicity. Sanger sequencing confirmed the variant in the index patient, while both parents tested negative, indicating a de novo mutation.

**Figure 4:**
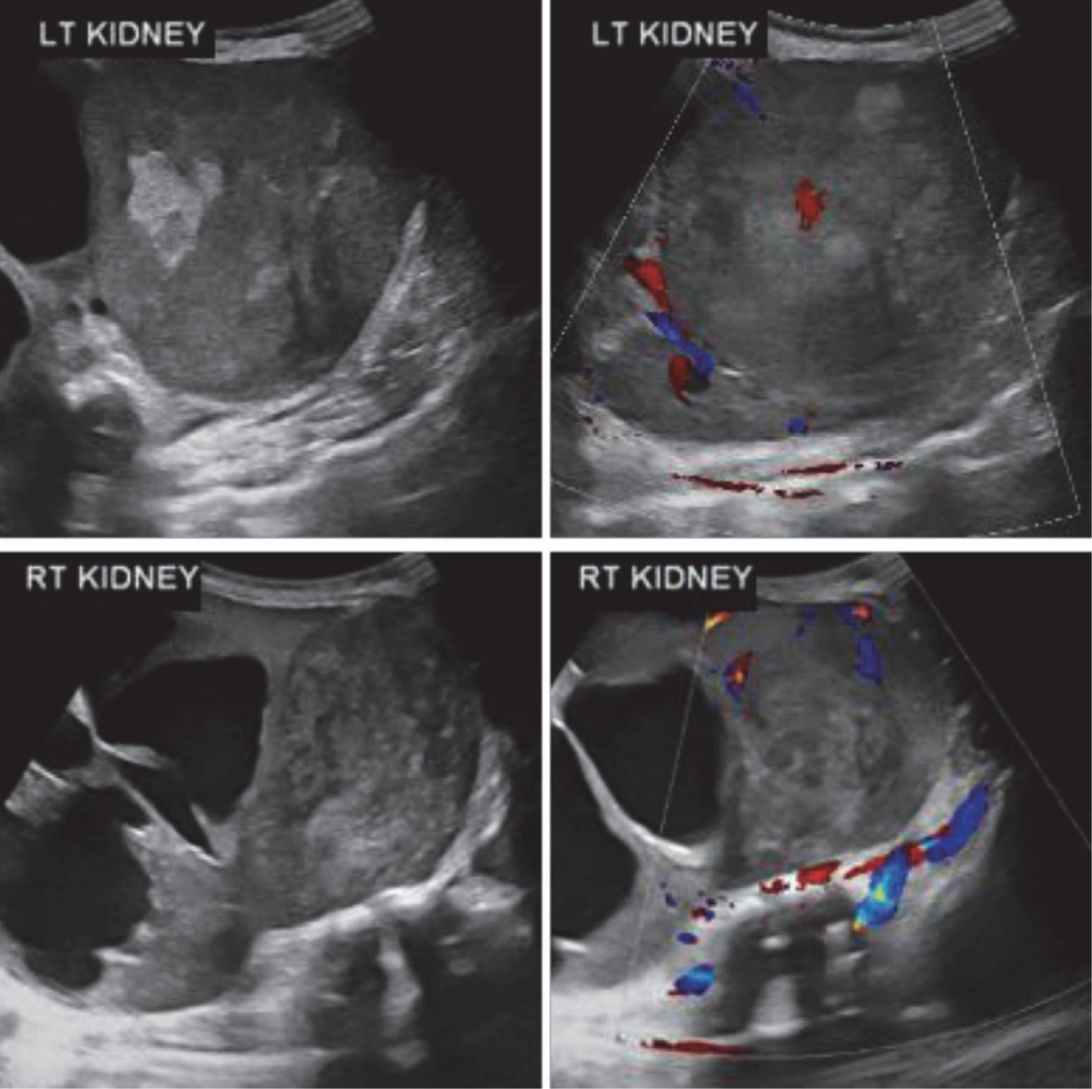
Renal ultrasound showing bilateral large lower pole renal masses crossing the midline. The masses show central hypoechoic/necrotic components and minimal internal vascularity. They are causing mass effect on the pelvicalceal system and subsequent hydronephrosis (Case 2).

**Figure 5:**
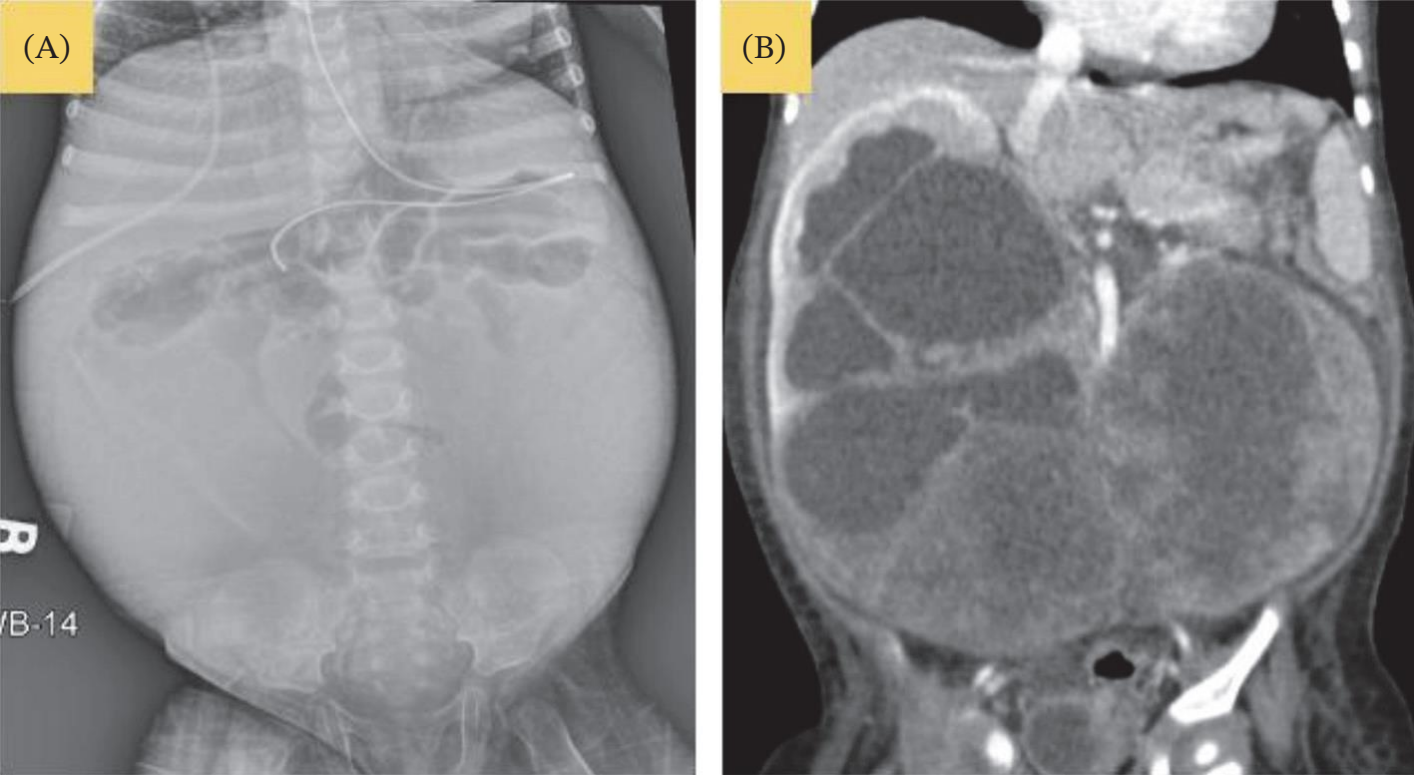
(A) Frontal abdomen radiograph shows central and superior displacement of the aerated bowel loops. Large soft tissue density is noted in the right and left abdomen, which is related to the enlarged horseshoe kidney. (B) An enhanced CT scan of the abdomen in coronal view shows bilateral renal enlargement with cross‐fusion of the lower renal poles, representing horseshoe kidney. Bilateral lower renal poles demonstrate large heterogeneous masses. Right hydronephrosis is also appreciated (Case 2).

Initial renal ultrasound (Figure 4) revealed large bilateral lower pole masses with hypoechoic, likely necrotic centers and minimal internal vascularity. A contrast‐enhanced abdominal CT scan (Figure 5) confirmed the presence of a horseshoe kidney with bilateral heterogeneously enhancing lower pole masses.

A renal biopsy showed spindle cells and primitive tubules, consistent with Stage IV aggressive Wilms tumor. The patient was initiated on chemotherapy, including doxorubicin. However, follow‐up imaging after the initial chemotherapy cycles revealed progressive tumor growth, indicating treatment resistance. Given the poor response, she underwent bilateral radical nephrectomy with peritoneal dialysis catheter insertion.

Postoperatively, the patient was admitted to the pediatric intensive care unit (PICU) with respiratory failure secondary to aspiration pneumonia, hypertension, and sepsis (evidenced by fever, leukocytosis, and elevated inflammatory markers). She was intubated and received broad‐spectrum antibiotics (meropenem, vancomycin, voriconazole, and levofloxacin), sedatives (midazolam, fentanyl, and dexmedetomidine), IV dextrose, and inotropic support. Her condition gradually stabilized, and she was successfully weaned off inotropes and discharged several weeks later.

Approximately 1 month after discharge, she was readmitted with high‐grade fever and hypotension. Septic shock was suspected, particularly in the context of underlying chemotherapy‐induced cardiomyopathy. A partial septic workup showed elevated WBC and inflammatory markers. She was treated empirically with ceftazidime, meropenem, and vancomycin. Cultures from blood and peritoneal fluid were negative. Her condition improved, and she was discharged.

Unfortunately, in the weeks that followed, the patient was readmitted with respiratory failure and multiorgan dysfunction. Despite supportive care, she deteriorated. After discussions with the medical team and family, care was transitioned to comfort measures, and a do not attempt resuscitation (DNAR) order was enacted. The patient passed away at 21 months of age.

## 3. Discussion

DDS presents a complex and variable clinical spectrum, driven largely by mutations in the *WT1* gene. As shown in our two cases, the phenotypic heterogeneity of DDS necessitates personalized approaches to diagnosis and management. Both patients demonstrated distinct clinical trajectories; one with early nephropathy and ambiguous genitalia without tumor development, and the other with rapid onset of Wilms tumor and no external genital abnormalities, underscoring the disease’s variability [1, 4, 5], even within the same genetic umbrella.

Both of our patients harbored nonsense variants resulting in premature termination of the WT1 protein, although the variants occurred in different functional regions of the gene. Variants affecting different domains of WT1 may produce variable functional consequences and likely contribute to the phenotypic heterogeneity observed in WT1‑related disorders.

Importantly, neither case had a positive family history of DDS or related disorders, which is consistent with the typically sporadic nature of *WT1* mutations [2]. Early identification and management of DDS are essential in improving patient outcomes [1, 4, 5]. In our first case, despite early signs of nephropathy and ambiguous genitalia, the progression to renal failure was complicated by intussusception and treatment delays due to the COVID‐19 pandemic. Delayed intervention can exacerbate disease progression, reinforcing the importance of prompt genetic testing and clinical evaluation in suspected DDS cases [2]. As noted by Lopez‐Gonzalez and Ariceta [14], *WT1*–related disorders encompass a broader spectrum beyond classic DDS, with varying degrees of nephropathy and genital anomalies, further complicating early diagnosis.

DDS is classically associated with a triad of nephrotic syndrome, ambiguous genitalia, and Wilms tumor [1]. However, not all patients develop all three features [2]. WT1 mutations, depending on their location and impact on protein function, are associated with variable clinical outcomes [15]. For example, truncating mutations in exons 8 and 9, such as c.1384C >T (p.Gln462Ter) seen in our Case 1, are frequently associated with early‐onset nephropathy and genital anomalies Truncating mutations located in the zinc‐finger DNA–binding domain of WT1 (exons 8–10), such as c.1384C >T (p.Gln462Ter) identified in our first patient, have been reported in association with steroid‐resistant nephrotic syndrome (NPHS4) and early‐onset renal disease. but may not always result in tumor development [16, 17]. In contrast, the variant c.453G >A (p.Trp151Ter) identified in Case 2 occurs in the N‑terminal regulatory region of WT1 and has been linked to a higher likelihood of Wilms tumor development and rapid progression to ESRD [18].

This phenotypic variability has led researchers to explore the molecular underpinnings of why some DDS patients develop Wilms tumor while others do not. One proposed mechanism involves the degree of residual WT1 protein function and how it affects the regulation of genes involved in nephrogenesis. Additionally, alternative splicing disruptions caused by intronic variants, as highlighted by Inoue et al. [19] may also contribute to clinical diversity. It is, therefore, plausible that both coding and noncoding variants in *WT1* influence tumor susceptibility, although further studies are needed to confirm these associations.

In our Case 2, the presence of a horseshoe kidney, bilateral renal masses, and eventual diagnosis of Stage IV Wilms tumor illustrates the importance of ongoing tumor surveillance in DDS patients, even in the absence of external genital anomalies.

The management of DDS remains a clinical challenge. The decision to proceed with bilateral nephrectomy is often weighed against the need to preserve renal function. Some experts advocate for prophylactic nephrectomy before Wilms tumor develops, especially in patients with high‐risk *WT1* variants, while others prefer to delay surgery until disease progression mandates it [1, 4, 5]. Both of our cases unfortunately ended in mortality, despite intensive interventions including chemotherapy, nephrectomy, and dialysis, highlighting the ongoing need for improved prognostic tools and individualized management plans.

Renal transplantation offers the best long‐term outcome in DDS patients who progress to ESRD. Gariépy‐Assal et al.[1] and Rudin et al. [20] both emphasize the importance of minimizing dialysis duration prior to transplant, which is associated with improved survival and quality of life. Early transplant planning, particularly in centers equipped for pediatric transplants, should be integrated into the long‐term management strategy.

Multidisciplinary care is paramount in DDS, involving pediatric nephrology, oncology, endocrinology, urology, genetics, and specialists with expertise in DSD. Additionally, patients with ambiguous genitalia require sensitive and family‐centered counseling and lifelong surveillance due to the elevated risk of gonadal malignancy [13].

Thus, advances in molecular diagnostics may allow for earlier detection and more refined genotype–phenotype predictions. Moreover, the emergence of targeted therapies, for example, agents that modulate *WT1*–regulated pathways or correct splicing defects, offers hope for more effective and less toxic treatments. Integration of NGS and prenatal genetic screening could also facilitate earlier intervention in high‐risk families or populations.

In conclusion, these two cases highlight the spectrum of DDS presentations and underscore the urgent need for earlier genetic diagnosis, tailored management, and collaborative multidisciplinary care. As our understanding of the molecular basis of DDS continues to evolve, future therapeutic strategies may become more personalized, improving both survival and quality of life in these vulnerable pediatric patients.

## Author Contributions

**Afaf Alsagheir:** conceptualization, supervision, writing – reviewing and editing. **Waleed AL-Amoudi**, **Abdulrahman Alnwiji, and Megren S. Alqarni**: data curation, investigation, writing – original draft preparation. **Raghad Alhuthil and Sarah Murad:** methodology, validation, and writing – original draft preparation.

## Funding

The authors have nothing to report.

## Disclosure

All authors read and approved the final manuscript.

## Ethics Statement

This study was approved by the Research Ethics Committee at King Faisal Specialist Hospital and Research Centre (Reference Number 2231110).

## Consent

Written informed consent was taken for the genetic studies.

## Conflicts of Interest

The authors declare no conflicts of interest.

## Data Availability

The data that support the findings of this study are available from the corresponding author upon reasonable request.
